# Modeling the Impact of Integrating HIV and Outpatient Health Services on Patient Waiting Times in an Urban Health Clinic in Zambia

**DOI:** 10.1371/journal.pone.0035479

**Published:** 2012-04-23

**Authors:** Sarang Deo, Stephanie M. Topp, Ariel Garcia, Mallory Soldner, Kezban Yagci Sokat, Julien Chipukuma, Chibesa S. Wamulume, Stewart E. Reid, Julie Swann

**Affiliations:** 1 Indian School of Business Hyderbad, India; 2 Centre for Infectious Disease Research in Zambia, Lusaka, Zambia; 3 Nossal Institute for Global Health, University of Melbourne, Melbourne, Australia; 4 Stewart School of Industrial and Systems Engineering, Georgia Institute of Technology Department, Atlanta, United States of America; 5 Industrial Engineering and Management Sciences, Northwestern University, Evanston, Illinois, United States of America; 6 Lusaka Health District, Lusaka, Zambia; 7 Department of Medicine, University of Alabama at Birmingham, Birmingham, Alabama, United States of America; Vanderbilt University, United States of America

## Abstract

**Background:**

Rapid scale up of HIV treatment programs in sub-Saharan Africa has refueled the long-standing health policy debate regarding the merits and drawbacks of vertical and integrated system. Recent pilots of integrating outpatient and HIV services have shown an improvement in some patient outcomes but deterioration in waiting times, which can lead to worse health outcomes in the long run.

**Methods:**

A pilot intervention involving integration of outpatient and HIV services in an urban primary care facility in Lusaka, Zambia was studied. Data on waiting time of patients during two seven-day periods before and six months after the integration were collected using a time and motion study. Statistical tests were conducted to investigate whether the two observation periods differed in operational details such as staffing, patient arrival rates, mix of patients etc. A discrete event simulation model was constructed to facilitate a fair comparison of waiting times before and after integration. The simulation model was also used to develop alternative configurations of integration and to estimate the resulting waiting times.

**Results:**

Comparison of raw data showed that waiting times increased by 32% and 36% after integration for OPD and ART patients respectively (p<0.01). Using simulation modeling, we found that a large portion of this increase could be explained by changes in operational conditions before and after integration such as reduced staff availability (p<0.01) and longer breaks between consecutive patients (p<0.05). Controlling for these differences, integration of services, per se, would have resulted in a significant decrease in waiting times for OPD and a moderate decrease for HIV services.

**Conclusions:**

Integrating health services has the potential of reducing waiting times due to more efficient use of resources. However, one needs to ensure that other operational factors such as staff availability are not adversely affected due to integration.

## Introduction

The human immunodeficiency virus (HIV) epidemic remains a major global public health challenge, with a total of 33.4 million people living with HIV worldwide, 2.7 million people newly infected in 2008, and 5.25 million people receiving antiretroviral therapy in low- and middle-income countries. In sub-Saharan Africa alone, the absolute number of people receiving life-long treatment increased by over 1 million, from 2,950,000 in 2008 to 3,910,000 by the end of 2009 [1]. Creating practical and sustainable systems to provide care for this growing population is one of the most pressing challenges facing health care planners and policy makers in resource limited settings today.

Many primary healthcare services in sub-Saharan Africa are delivered through *vertical* systems, where services for tuberculosis, routine outpatient care, maternal and child health and family planning are co-located but use separate physical space, staff and medical records [2], [3]. This fragmentation of service delivery has been further accentuated in countries that have recently experienced rapid scale-up of antiretroviral therapy (ART) programs [4], [5], [6] for HIV-infected individuals. This vertical approach to scaling up HIV services has facilitated quick establishment and quality-assured implementation of a complicated medical service in high prevalence settings with typically weak service delivery systems [7]. Nonetheless, concerns have been raised regarding the long-term feasibility and sustainability of these separate services [8], [9], [10], whose rapid growth has strained coverage and quality of existing primary health care services [7], [11].

Recognizing the limitations of vertical service models, a small number of pilots have been initiated recently seeking to integrate HIV and non-HIV care at the primary care level [12], [13], [14], [15], [16], [17], [18]. Definitions of ‘integrated’ services and the extent of service-integration reported in this nascent literature is varied, ranging from paper referral systems linking physically separate services, to services delivered in the same location and by the same cadres of healthcare workers in a facility. In one such initiative, undertaken by co-authors of this paper, integration was defined as the harmonization of all point-of-care services including registration, medical record keeping, patient flow, and dispensing services. The feasibility of this model during the pilot phase in two clinics has been previously reported [12] and the scale-up of the model to a further seven clinics (nine in total) resulted in a doubling of clinic-based uptake of HIV counseling and testing amongst outpatients not already enrolled in HIV treatment [12].

Despite these accomplishments, a limitation of this integrated service delivery model was an increase in waiting times for all outpatients, including those enrolled in HIV care and treatment as well as those seeking non-HIV services [12], [19]. Since waiting time experienced by patients has been shown to adversely affect their health seeking behavior [20], [21] and treatment adherence [22], increased waiting times were perceived to be a barrier to, or at least limitation of, scaling up this service-delivery model.

However, it is conceivable that this increase in waiting time could be due to changes in operational conditions that are not related to integration per se, such as patient load, patient mix, and staff availability. In this paper, we employ advanced operations research techniques in conjunction with detailed operational data from the original dataset of the integration pilot to achieve following objectives: (i) disentangle the relative impact of the integration of services, i.e., the sharing of resources across HIV and outpatient clinics, and other confounding factors on the increase in waiting time, and (ii) identify alternative operational configurations of integration that lead to reductions in waiting times.

## Methods

### Ethics Statement

This study was approved by the Research Ethics Committee of the University of Zambia and the Institutional Review Board at the University of Alabama at Birmingham. Individual informed consent was not obtained since all data were analyzed anonymously.

### Study setting

This study was conducted at an urban clinic in Lusaka, Zambia. The study site was the first clinic to participate in a pilot program to integrate HIV antiretroviral treatment department (ART clinic) and non-HIV outpatient department (OPD clinic) in urban Lusaka clinics. The daily patient load, calculated from attendance figures recorded in the clinic's registers, was approximately 80 (50 OPD patients and 30 ART patients). Average staffing levels at the clinic per five-hour shift across both departments comprised 3–4 nurses and 1–3 clinicians (clinical officers and physicians). Additionally, 2–3 peer educators trained in psychosocial counseling and carrying out non-clinical tasks worked in the ART clinic. The OPD clinic was open 24 hours a day and operated in three shifts (8 am to 1 pm; 1 pm to 6 pm; 7 pm to 7 am). The ART clinic operated in a single shift from 8 am to 2 pm.

Before the introduction of the integrated service delivery model, the OPD clinic provided pay-for-service (with exceptions for patients with some chronic conditions including tuberculosis, asthma, chronic heart conditions and epilepsy), episodic, general medical care to any presenting patient. The ART clinic provided free chronic care to any HIV-infected patient who requested enrollment [6] including HIV-infected patients not yet clinically eligible for antiretroviral drugs (ARVs). While both OPD and ART clinics are Ministry of Health services, the ART clinic received significant additional financial and technical support from international donors such as the U.S. government's President's Emergency Plan for AIDS Relief (PEPFAR) through partnering NGOs such as the Centre for Infectious Disease Research in Zambia (CIDRZ).

### Integration

Starting in September 2007, the Lusaka District Health Management Team (LDHMT) initiated a pilot to integrate OPD and ART clinics in two urban Lusaka sites. Specifically, in the site studied here, integration was initiated in the week starting July 14, 2008. The model and process of integration has been described in detail elsewhere [12]. Briefly, it involved harmonizing the patient flow for OPD and ART such that patients were seen in a first come, first served manner irrespective of presenting complaint (with the exception of medical emergencies). Modifying physical space and cross-training of staff took place before integration along with a program of community sensitization to HIV/AIDS involving drama performances and door-to-door visits to inform the catchment population about impending changes to clinic services.

**Figure 1:**
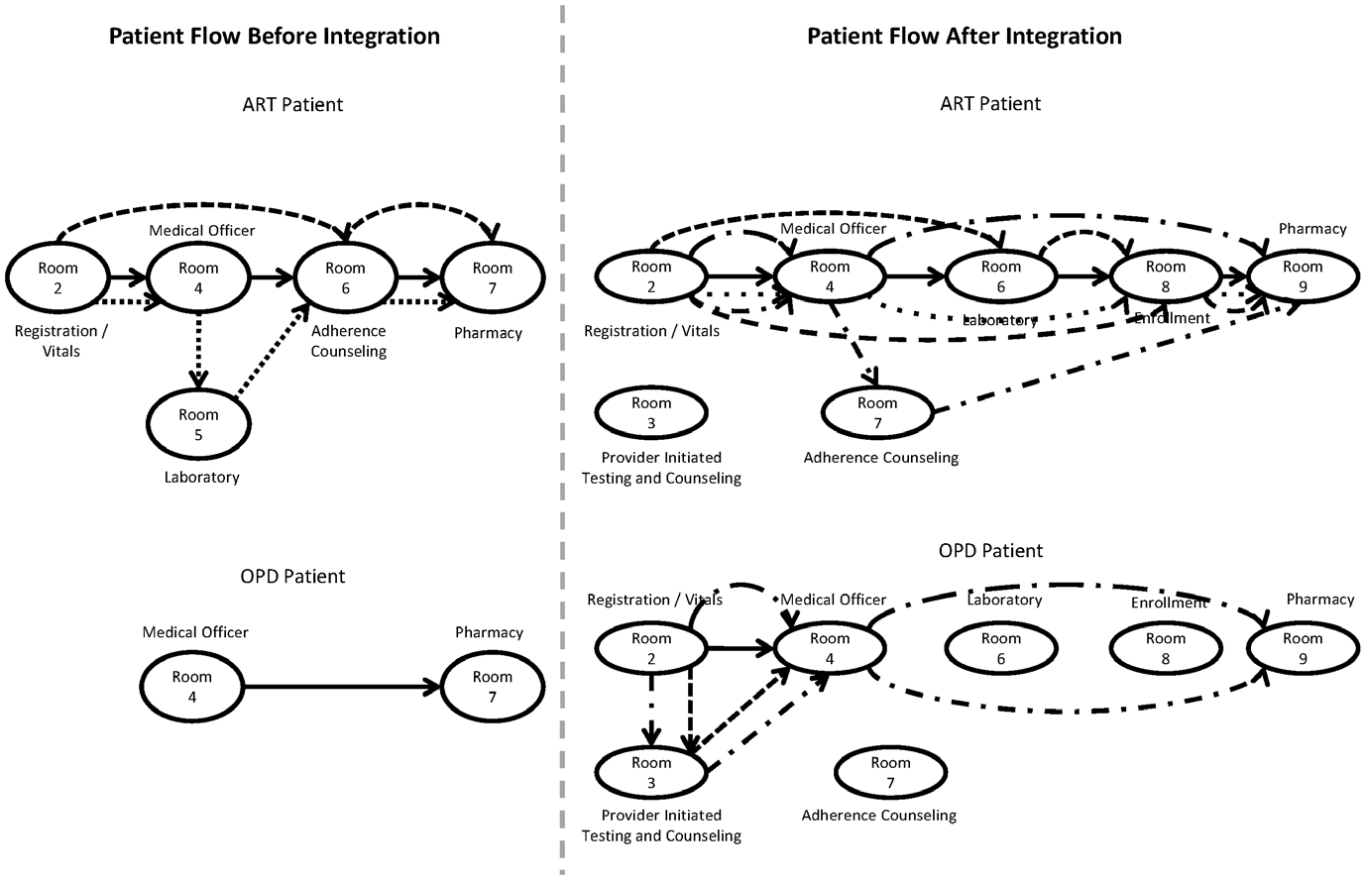
Typical patient flows before and after integration. Dotted lines represent different flow patterns through the clinics.

Integration of services resulted in no substantive changes to processes of clinical care for ART patients. However, the integrated model included the addition of two service steps for OPD patients compared to the non-integrated service; first, the measurement and recording of patient vital signs (including weight, blood pressure and temperature) and second, the offer of provider-initiated HIV testing and counseling (PITC). Both these steps in the patient flow occur *prior* to a patient being screened by a clinician (**Figure 1**).

### Time and Motion Study

We conducted a time and motion study over two, seven-day periods (one month before integration and six months after integration) during the busiest clinic hours of 7:30 am to 12:00 noon. Specifically, the pre-integration data were collected from June 23^rd^ to 29^th^ 2008 and post-integration data were collected from January 19^th^ to 25^th^ 2009. In each instance, we attached a form to patients' medical files to record the time of patient arrival and the start and end times of patient interaction at each clinical station for OPD and ART patients (vitals, triage, screening room, laboratory, pharmacy, ART adherence, ART enrollment) during the patient flow process. Two of the co-authors recorded the time of patient arrival in the clinic while the times at subsequent stations through the clinic were recorded by the respective healthcare workers attending the patients. The difference between a patient's start and end times at each step was defined as his/her process time whereas the difference between the end time at one step and the start time at the subsequent step was defined as his/her waiting time for the next step. We assumed that a patient's whole stay in a room was part of the processing or consultation time and that the worker was idle between the end time of the previous patient and the start time of the subsequent patient. Hence, any time spent by the nurses with charts during a patient's stay in the room was assumed to be a part of the process time of that particular patient. We also detected idle times from the workers corresponding to having no patients inside the room. Such times were defined as break times. Due to limited number of resources in the clinic, we noticed that staff availability per room changed during clinic hours. In order to account for this variability, we looked at the number of resources available per each room for different time intervals such as 60 minutes, 30 minutes and 15 minutes. We decided to use 15 minutes intervals since it represented the fluctuations the best.

### Statistical analysis

We conducted t-tests to compare process time and waiting time before and after integration for each patient type. We also conducted t-tests to compare the length of breaks before and after integration taken by healthcare workers between consecutive patients. Further, we conducted paired t-tests to compare operational factors in the two observation periods: total patient load, mix of patients between OPD and ART services, availability of staff at each station and the number of rooms visited during the patient flow process before and after integration. For total patient load, mix of patients between OPD and ART services, and number of rooms visited, we paired hourly observations before and after integration. For staff availability in each room, we paired observations over intervals of 30 minutes. All statistical analyses were performed using SAS/STAT software, Version 9.1 (Cary, NC, USA).

### Simulation

Simulation was chosen to control for the differences in operational characteristics before and after integration, isolate the effects of the integration, and examine alternate designs to the system. We used the observations from the time and motion study to construct patient flow process diagrams (**Figure 1**) for ART and OPD patients before and after integration. We then developed three base models of discrete event simulation (DES) representing the OPD clinic and ART clinic before integration and the combined clinic after integration respectively. We used ARENA® (Rockwell Automation, Milwaukee, WI, USA) for all simulation modeling and analysis. DES was chosen rather than system dynamic modeling because it allowed incorporation of variability and fit the data well.

#### Development and validation of base models

Data from the time and motion study was used to fit probability distributions for the following key inputs in the simulation model: (i) arrival rates representing typical weekdays with three blocks per day for each patient type using Poisson arrivals, (ii) routes representing the four most common patterns of patient flows, each comprising different sets of rooms, (iii) resources representing the average number of staff for each room scheduled in 15 minute intervals, (iv) process times using distributions specific to room and patient type, (v) break times with distributions, truncated at 15 minutes, to represent either physical break or time used for documentation by staff. We ran 200 replications of the simulation model and the length of each replication was one working day at the clinic. The distribution of the waiting times obtained from pre- and post-integration simulation models were statistically compared against actual data collected from the time and motion study for validation.

#### Isolating the impact of integration

To isolate the impact of integrating HIV and outpatient services from that of changes in other operational conditions such as patient load, patient mix, staff availability etc., we created two variants of the post-integration DES model and populated it with input data from before integration. In the first variant, healthcare workers were completely integrated but additional services (registration and vitals and counseling and testing) were not provided to the OPD patients. The second variant included the two additional steps (registration and HIV testing) for the OPD patients, which were conducted by three additional health workers, in accordance with the actual practice. Similarly, in accordance with the quantity measured in the time and motion data during the post-integration phase, we assumed that 30% of the OPD patients accepted PITC.

#### Alternative integration configurations

We conducted simulation experiments to evaluate alternative models of integration and their impact on patient waiting times.

Experiment I (Additional resources for added steps): While we acknowledge the difficulties in providing additional human resources, our objective was to estimate the number of resources needed to maintain waiting times for each patient group at their levels before integration. We analyzed the impact of adding one more healthcare worker at the registration/vitals and PITC step on waiting time.

Experiment II (Steps to Integrate): In many settings, where it might not be feasible to integrate all steps in the care delivery process, a natural question is: which process steps would provide the maximum reduction in waiting time from integration? We constructed scenarios of partial integration where either the clinician or the pharmacy step could be integrated or not since these two steps resulted in the greatest impact in the waiting time. In all of these scenarios, the registration/vitals step was always integrated.

Experiment III (Impact of ART vs. OPD patient ratio): We analyzed the impact of patient mix on waiting times after integration because the patient mix can differ by clinic. We simulated scenarios with ART to OPD patient ratios ranging from 0% to 100% in increments of 10% for both before integration and after integration models for a fixed number of total patients.

Experiment IV (Percentage of OPD patients tested): Different clinics might have different PITC uptake rates, depending on patient attitude and staff involvement, which consequently will alter the patient flow. We simulated these scenarios by varying the fraction of OPD patients accepting PITC from 0% to 100% in increments of 10%.

## Results

### Characterization of patient flow before and after integration

Figure 1 displays the main routes taken by ART and OPD patients before and after integration. The corresponding composition of routes for the two observation periods is shown in Table 1. These data highlight the complexity of the patient flow and service operations conducted in the ART and the integrated clinics

**Table 1:** Composition of patient routes before and after integration.

ART Patients	Before integration (N = 155)	After integration (N = 125)
Registration/Vitals, Medical Officer, Adherence Counseling, Pharmacy	31%	
Registration/Vitals, Medical Officer, Laboratory, Adherence Counseling, Pharmacy	20%	
Registration/Vitals, Adherence Counseling, Pharmacy	12%	
Registration/Vitals, Adherence Counseling, Medical Officer, Pharmacy	8%	
Registration/Vitals, Medical Officer, Pharmacy	5%	
Others*	24%	
Registration/Vitals, Medical Officer, Adherence Counseling, Pharmacy		25%
Registration/Vitals, Medical Officer, Adherence Counseling,		13%
Registration/Vitals, Adherence Counseling, Pharmacy		12%
Registration/Vitals, Adherence Counseling		10%
Registration/Vitals, Medical Officer, Pharmacy		7%
Registration/Vitals, Pharmacy		6%
Registration/Vitals, Medical Officer,		5%
Others**		22%

OPD Patients	Before integration (N = 155)	After integration (N = 125)
Medical Officer, Pharmacy	90%	
Medical Officer	8%	
Others∧	2%	
Registration/Vitals, Medical Officer, Pharmacy		50%
Medical Officer, Pharmacy		16%
Registration/Vitals, PITC, Medical Officer, Pharmacy		12%
Registration/Vitals, Medical Officer		12%
Registration/Vitals, PITC, Medical Officer		4%
Others∧∧		6%

* (Registration/Vitals, Adherence Counseling), (Registration/Vitals, Pharmacy), (Registration/Vitals, Registration/Vitals, Medical Office, Adherence Counseling, Medical Officer, Laboratory, Pharmacy).

** (Registration/Vitals, Medical Officer, ART enrollment, Adherence Counseling, Pharmacy), (Registration/Vitals, Laboratory, Adherence Counseling).

∧(Medical Officer, Tuberculosis), (Medical Officer, Pharmacy, Tuberculosis).

∧∧Registration/Vitals, PITC, Pharmacy), (Registration/Vitals, Adherence Counseling, Tuberculosis).

### Raw Data Analysis

Before integration, an average ART patient spent 114.99 minutes in the clinic. Out of this, the service time (time spent in registration, with a clinical officer, and in pharmacy) was 21.76 while waiting time was 93.23 minutes. Similarly, an OPD patient spent 90.8 minutes in the clinic, of which 7.01 minutes was the process time and 83.79 minutes was waiting time. The average process time for ART patients was higher than those for OPD patients before integration (21.76 vs. 7.01; p<0.001). Total time spent in the clinic increased after integration for both ART patients (121.31 vs. 90.8; p<0.01) and OPD patients (110.58 vs. 83.79; p<0.01). There was a significant increase in waiting time for both ART patients (127.15 vs. 93.23; p<0.01) and OPD patients (110.58 vs. 83.79; p<0.01). This increase was partly driven for by an increase in service times (ART patients – 26.65 vs. 21.76; p<0.01, OPD patients – 10.73 vs. 7.01, p<0.01). Service time increased in the registration step for both ART (10.65 vs. 7.38, p<0.01) and OPD patients (2.71 vs. 0.00). The service time in pharmacy and with the clinical officer was not significant for both ART and OPD patients. These results are summarized in Table 2.

**Table 2:** Raw comparison of average total time, waiting time and process times at different steps (minutes) spent by ART and OPD patients before and after integration.

	Before	After	p-value
ART Patients			
Total time	115	154	<0.01
Total waiting time	93	127	<0.01
Total process time	22*	27**	<0.01
- Registration	7	11	<0.01
- Clinical Officer	10	12	0.17
- Pharmacy	4.03	4.37	<0.01
OPD Patients			
Total time	91	121	<0.01
Total waiting time	84	111**	<0.01
Total process time	7*	11	<0.01
- Registration	-	3	NA
- Clinical Officer	4	5	<0.01
- Pharmacy	3	3	0.40

* Total process time for ART patients was higher than the OPD patients before integration (21.76 vs 7.01; p<0.01).

** Total process time for ART patients was higher than the OPD patients after integration (26.65 vs 10.03; p<0.01).

The increase in waiting time could be partly attributed to potential confounding due to the substantial differences in operational factors in the short observations periods before and after integration. Staffing hours were significantly lower post-integration with the largest reduction occurring in pharmacy. Also, “breaks” between patients were longer after integration for both ART and OPD patients. The hourly arrival rate, hourly ART patient ratio, hourly OPD patient ratio and ART process time did not change significantly (Table 3).

**Table 3:** Comparison of various operational factors before and after integration.

Factor	Before	After	p –value
Hourly Patient Arrival Rate	15	17	0.24
Hourly ART Patient Ratio	0.27	0.19	0.08
Hourly OPD Patient Ratio	0.73	0.81	0.08
ART Total Process Time (minutes)	2819	2819	0.47
OPD Total Process Time (minutes)	7	11	<0.01
Complexity (Number of rooms visited)	2.42	2.73	0.02
Average Number of Human Resources Available at Registration (ART only)	1.64	0.78	<0.01
Average Number of Human Resources Available at Clinical Officer	1.74	1.27	<0.01
Average Number of Human Resources Available at Pharmacy	1.91	0.67	<0.01
Average Length of Break between ART Patients at Clinical Officer (minutes)	2.46	8.45	<0.01
Average Length of Break between OPD Patients at Clinical Officer (minutes)	2.02	2.8	0.05
Average Length of Break between OPD Patients at Pharmacy (minutes)	2.53	4.2	<0.01

### Validation of Simulation results

Statistical tests showed that the raw data for average waiting times was within the 95% confidence interval of the simulation output of their corresponding scenarios (Table 4).

**Table 4:** Validation of the simulation models (comparison of simulation output with the results of the time and motion study).

	Time and motion study results (minutes)		Simulation results (minutes)		
	Average	Std Dev	Average	Lower 95%	Upper 95%
1. Pre-integration ART	93	49	86	76	96
2. Pre-integration OPD	84	47	85	80	90
3. Post-integration ART	127	51	128	118	139
4. Post-integration OPD	111	46	117	108	126
5. Post-integration Overall	113	48	121	112	131

### Isolating the impact of integration

Scenarios 0 and 1 in Table 5 show that the waiting time should have decreased for both types of patients in the absence of the added step of PITC for OPD patients and with inputs from before integration. However, comparing scenarios 1 and 2 shows that the addition of PITC and registration for OPD patients (an increase of 4.36 minutes of process time), even with a corresponding increase in staffing for PITC and registration/vitals, significantly increased the waiting times for OPD patients (83 minutes) and also for ART patients (25 minutes). Similarly, comparing scenarios 2 and 3 highlights that a substantial portion of the increase in waiting times could be attributed to adverse operational conditions after integration such as low staff availability, high patient load, a more complex patient mix.

**Table 5:** Accumulated waiting times (minutes) in new Discrete Event Simulation Models after adjusting for additional steps and other changes in operational factors before and after integration (where PITC indicates Provider Initiated HIV Testing and Counseling).

Scenarios	ART Patient Accumulated Waiting Time	OPD Patient Accumulated Waiting Time
0. Pre-integration (with pre-integration parameters, no PITC and no added resources)	86	85
1. Post-integration (with pre-integration parameters, no PITC and no added resources)	80	53
2. Post-integration (with pre-integration parameters, PITC and 3 additional resources)	105	136
3. Post-integration (with post-integration parameters, PITC and 3 additional resources)	128	117

### Alternative integration configurations

Experiment I (Number of resources for added steps): Including four healthcare workers for registration and PITC (i.e., one more than the current practice) would result in waiting times at or below pre-integration levels for ART patients (76 minutes vs. 93 minutes) and OPD patients (80 minutes vs. 83 minutes) in spite of adverse operating conditions.

Experiment II (Steps to Integrate): If only clinical officer step is integrated, ART and OPD waiting times are 109 minutes and 139 minutes respectively. If only pharmacy step is integrated, ART and OPD waiting times are 108 minutes and 140 minutes respectively. These are not substantially higher than when both steps are integrated – 105 minutes and 136 minutes for ART and OPD respectively. This underlines the attractiveness of partially integrated scenarios if complete integration cannot be achieved due to constraints in cross-training some categories of healthcare workers.

**Figure 2:**
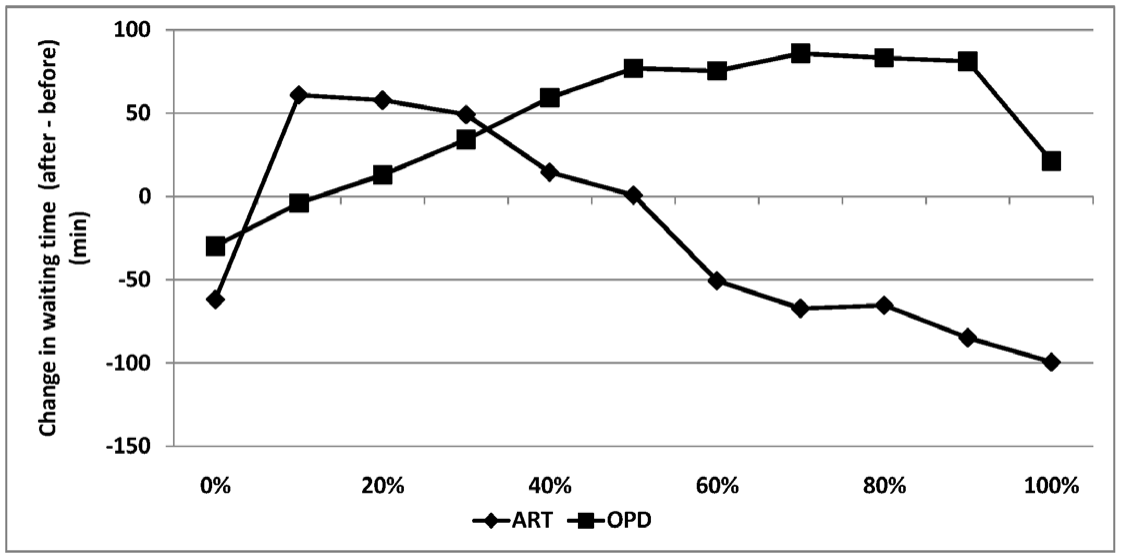
Impact of ART to OPD patient ratio on difference in waiting times before and after integration. Positive numbers denote an increase in waiting time whereas negative numbers indicate a reduction in waiting time due to integration. Zero denotes that the waiting times before and after integration are equal.

Experiment III (Impact of ART vs. OPD patient ratio): As the ART patient volume increases beyond 30% (our baseline), the waiting time for ART patients decreases but the waiting time for OPD patients increases (Figure 2). In fact, the post-integration waiting time for ART patients would be lower than pre-integration levels in clinics that have 50% or more ART patients (Figure 2).

**Figure 3:**
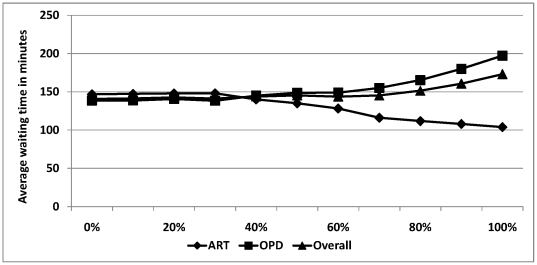
Average waiting time of ART patients, OPD patients and overall in the post-integration clinic as a function of the fraction of OPD patients who accept PITC.

Experiment IV (Percentage of OPD patients tested): Keeping everything else fixed, the average waiting time for OPD patients would increase as more OPD patients accept PITC, but the average wait for ART patients would decrease (**Figure 3**).

## Discussion

This work was motivated by recent observations that a pilot integration of HIV and non-HIV services led to increased waiting time for patients [12]. Waiting time is an important operational determinant of health seeking behavior of patients [20], [21], is associated with reduced adherence to HIV treatment [22] and reduced patient satisfaction [12], [23]. Hence, we investigated the causes of the increase in waiting time and focused on isolating the relative impact of integration itself from that of changes in other operational conditions that might have coincided with integration.

A raw comparison of data before and after integration indicates an increase in waiting times but it is confounded by the fact that the two observation periods are short and characterized by substantial fluctuations in their operational conditions. Our results indicate that the unadjusted comparisons of raw data overestimate the increase in waiting times for OPD patients but underestimate the increase for ART patients. We find that one portion of the increase in waiting times is because of the increase in process times due to added steps that were intrinsic part of the integration such as registration and vitals, and provider initiated testing and counseling for OPD patients. Interestingly, addition of registration and vitals for OPD patients also increased registration process time for ART patients. This might be due to potential diseconomies of scope, i.e., health care workers slowing down while switching between two different types of patients.

The magnitude of increase in waiting times was significantly higher compared to the magnitude of increase in process times and break times. This can be explained using concepts of queuing theory, according to which waiting times can be substantially greater than the process times in service systems with substantial variability in arrival and service processes, especially if the utilization of resources is close to 100%.

Another substantial portion of the increase was because of healthcare workers taking longer breaks between successive patients after integration and due to lower staffing levels after integration. Operational experience of the authors suggests that reduced staff coverage and longer break times are not a direct result of integration. Primary healthcare clinics in this setting experience rapid and sometimes unpredictable changes in staffing levels and seasonally variable patient attendances, which can substantially affect in-clinic operations. Thus, while we cannot totally discount the possibility that integration *contributed* to absenteeism, we do note that variation in staff levels in Lusaka facilities occur in both integrated and non-integrated facilities related to inter-facility rotation, a high incidence of in-service training courses, and high rates of study, vacation and sick leave amongst healthcare professionals. Our study was not designed to uncover reasons behind longer breaks taken by health workers between patients but anecdotal evidence suggests that this could result from several factors, e.g. the mental switching time between different patient types and dissatisfaction with the rearrangement of responsibilities in the integrated system. Both these factors are likely to be present in any integration such system change, and policy makers could minimize the negative impact of integration on waiting times by addressing them preemptively during the planning and pre-training phase.

Overall, our findings from detailed analysis (as against a basic analysis of the raw data) indicate that an increase in waiting times should not be taken at face value and attributed to the integration itself, but rather that further analysis is needed to uncover the root causes of increases in waiting times and address them. Analysis of an alternative model of integrated service delivery, which did *not* include the addition of new processing steps (vitals collection and PITC for OPD patients), demonstrated that a clinic might be able to substantially reduce waiting times for both streams of patients by integration alone if steps are not added. This provides an additional rationale for integrating the two services in addition to the clinical motivations of improving continuum of care, strengthening HIV case finding and minimizing the negative psychosocial impact of isolating HIV and AIDS care and support services.

We also provide several tangible recommendations regarding the integration of clinics, where the recommendations can be broadly divided into two categories depending on the unit of analysis and level of decision-making. At the level of individual clinics, and while cognizant of the extreme human resource constraints in this setting, we suggest that prior to implementing this model of integrated service-delivery, policymakers and programmers consider where possible the addition of human resources for any additional steps (e.g. PITC). Careful consideration should also be given as to which steps in a patient care model are feasible to integrate and which should be left separate.

We find that small changes in staff availability for specific processes or in patient flow can have dramatic effects on patient waiting time. For instance, by increasing the number of staff conducting Vitals/Registration and PITC from three to four in the system studied, waiting times could be reduced to less than waiting times before integration. While, availability of additional staff is not guaranteed in resource- limited settings, conducting such an analysis ex-ante (instead of ex-post) provides policy makers with realistic targets for individual clinics, which could then be weaved into a district wide plan [24]. This finding also gives indirect support for task shifting, the provision of lay providers or peer educators to perform lower level tasks, in situations where there is a mechanism for hiring and training lower cost cadres of health care workers.

More interestingly, integration of only a *partial* set of clinic processes (only pharmacy or only the clinical officer) can also yield a significant portion of the reduction in waiting time obtained from complete integration. This has important implications for policymakers and programmers since it might not always be feasible to integrate all steps due to the prohibitive cost of cross-training healthcare workers and redesigning the entire physical layout of the clinics.

At the level of a health district, we highlight key characteristics such as the uptake of PITC and the OPD-ART patient ratio that might predispose some clinics to have more successful integrations than others (as measured by reduced patient waiting times). The findings in this area could be useful for District Health Management teams in prioritizing clinics that are most suitable candidates for integrated service delivery, or for identifying ones where waiting times may increase due to integration alone, where additional steps can be taken to ensure waiting times do not increase in those situations.

Holding available resources fixed before and after integration, clinics with a higher proportion of ART patients (>30% of total patients) were found to experience lower waiting times overall, with waiting times increasing for OPD patients but decreasing for ART patients. One potential driver for this result is that ART clinics in the urban Lusaka setting are (based on a staff to patient ratio analysis) typically better staffed than OPD clinics. Hence, integration could result in better sharing of resources that may not have been fully utilized for OPD patients before integration. This effect is less significant if OPD comprise a larger share of the patient pool.

Another clinic attribute that significantly affects waiting time in the integrated clinic is the proportion of OPD patients who accept PITC. We found, as expected, that the average waiting time for OPD patients increased as the PITC uptake increased since more patients require more services. However, interestingly, we also found that increased uptake of PITC in OPD reduced the average waiting time of ART patients. We hypothesize this is driven by the fact that patients not receiving the PITC “skip” to the clinical officer queue ahead of those receiving PITC, which is especially important because of heavy patient arrivals in morning hours. These patients not only avoid waiting in the PITC queue, but they also reach the clinician more quickly. Since the clinician is the most constrained resource of the clinic and since the queue for that room increases throughout the day, patients who reach it earlier are more likely to avoid the critical bottleneck.

A limitation of our study is that the data were gathered from a single urban clinic in the national capital, which may not have the same operational (e.g. proximity to other clinics and District officials) or epidemiological (e.g. urban versus rural population) characteristics as the other clinics throughout Zambia. Moreover, the data for this study was collected for one week before integration and one week after integration, staff knew the data were being recorded, and the data applies specifically to the model of integration implemented in this clinic. Our use of simulation modeling mitigates these limitations to some extent; simulation modeling is flexible enough to be able to create representative models for clinics with different staffing levels and patient characteristics. However, it still primarily considers the processing flow in the system we studied and is based on some assumptions associated with it, including that the operational changes are not associated with integration itself. If the observation periods were sufficiently long, comparison of raw waiting times would be sufficient but such research designs can be expensive and impractical due to their impact on routine care.

The study site was the first to be piloted under the LDHMT integration program. This likely contributed to organizational constraints that impacted the effectiveness of the implementation, including staff resistance to change, reduced morale due to operational uncertainty and requirement to do new tasks and time taken to adapt to new systems. We tried to mitigate this limitation by collecting the post-integration data six months after integration when some of these issues were brought under control through active partnership between LDHMT and the clinic leadership. Nonetheless, as the implementers learn from early integrations, some of these organizational issues may become less important in subsequent integrations. It could be important to replicate our findings in these facilities.

In this paper our data and analytical approach does not allow us to talk meaningfully about the ‘clinical experience’ of the patient. However, in a previous paper discussing the feasibility of integration in this and one other clinic, we reported that integration had no net effect on seasonal patient attendance rates during the first 6 months [12]. The same paper described qualitative data on patients' perceptions of clinical care following integration. These findings demonstrated an overwhelmingly positive perception by OPD patients resulting primarily from the routinization of vitals measurement, the introduction of PITC, and the more consistent availability of nursing staff. ART patients reported that integrated services were less stigmatizing by comparison to the separated services. Nonetheless both OPD and ART patients report the negative experience of increased waiting times [12].

The public health imperative to provide sustainable, affordable care and treatment services for HIV-infected population in the face of limited resources makes it critical to improve our understanding of the best way to strike a balance between clinical quality and operational efficiency. The underlying complexity of even the most basic primary care clinics, as highlighted in the data presented here, necessitates the use of a more rigorous approach to modeling and data analysis to understand the on-the-ground implications prior to implementation. Our findings demonstrate the value of applying operations research methods (e.g. simulation modeling) to thorny public health debates (e.g. vertical vs. integrated health systems) in resource-limited setting. In practice, even if full-scale modeling of this sort cannot be undertaken due to lack of resources and appropriate capabilities, post-integration data can be collected on key factors such as staffing resources and process times. If the former decreases or the latter increases, then integration may result in an increase in waiting times, that could then be reduced through appropriate management interventions. On a methodological front, our use of simulation modeling in conjunction with empirical analysis is novel and could be applied in other settings where longitudinal data collection is either prohibitively time consuming or expensive.
